# Supplementary material for: The Pathway to Cancer Cachexia: MicroRNA-Regulated Networks in Muscle Wasting Based on Integrative Meta-Analysis
Source: Int J Mol Sci. 2019 Apr 22;20(8):1962. doi: 10.3390/ijms20081962 (PMC6515458; doi:10.3390/ijms20081962)
Supplement: Supplementary file 1 [file ijms-20-01962-s001.zip › S1 Table.docx]

**Supporting Material**

**S1 Table. Gene List.**

List with ID, symbol, full name, and synonyms of the 52 differentially expressed genes validated in the skeletal muscles from cancer cachexia samples.

| ID | Official Symbol | Official Full Name | Synonyms |
| --- | --- | --- | --- |
| 11459 | Actc1 | actin, alpha 1 | Acts; Acta-2; Actsk-1 |
| 11513 | Adcy7 | adenylate cyclase 7 | AA407758 |
| 11450 | Adipoq | adiponectin, C1Q and collagen domain containing | Ad; APN; Acdc; apM1; adipo; Acrp30 |
| 654812 | Angptl7 | angiopoietin-like 7 | Angl7 |
| 494504 | Apcdd1 | adenomatosis polyposis coli down-regulated 1 | Drapc1 |
| 12176 | Bnip3 | BCL2/adenovirus E1B interacting protein 3 | Nip3; Bcl2 |
| 50908 | C1s1 | complement component 1, s subcomponent 1 | C1s; C1sa |
| 12323 | Camk2b | calcium/calmodulin-dependent protein kinase II, beta | KCC2B |
| 12389 | Cav1 | caveolin 1 | Cav; Cav-1 |
| 12609 | Cebpd | CCAAT/enhancer binding protein (C/EBP), delta | c/EBPdelta |
| 12845 | Comp | cartilage oligomeric matrix protein | TSP5 |
| 20315 | Cxcl12 | chemokine (C-X-C motif) ligand 12 | Pbsf; Sdf1; Tlsf; Tpar1; Scyb12 |
| 54709 | Eif3i | eukaryotic translation initiation factor 3, subunit I | Trip1; Eif3s2 |
| 14089 | Fap | fibroblast activation protein | SIMP ; SEPR |
| 67731 | Fbxo32 | F-box protein 32 | MAFbx; ATROGIN1; |
| 99571 | Fgg | fibrinogen gamma chain | FIBG |
| 56458 | Foxo1 | forkhead box O1 | Afxh; FKHR; Fkhr1; Foxo1a |
| 14313 | Fst | follistatin | FS |
| 57436 | Gabarapl1 | gamma-aminobutyric acid A receptor-associated protein-like 1 | GECI; Apg8l; Atg8l; GBRL1 |
| 15239 | Hgs | HGF-regulated tyrosine kinase substrate | tn; Hgr; Hrs |
| 66847 | Hint3 | histidine triad nucleotide binding protein 3 | HINT-3; HINT-4 |
| 15439 | HP | haptoglobin | HP-1; hpt; preHP2 |
| 15483 | Hsd11b1 | hydroxysteroid 11-beta dehydrogenase 1 | DHI1 |
| 15516 | Hsp90ab1 | heat shock protein 90 alpha, class B member 1 | Hsp84; Hsp90; Hspcb; Hsp84-1 |
| 16477 | Junb | jun B proto-oncogene | MyD21 |
| 80334 | Kcnip4 | Kv channel interacting protein 4 | Calp; KChIP4; Calp250; KChIP4a |
| 16773 | Lama2 | laminin, alpha 2 | dy; mer; merosin |
| 17260 | Mef2c | myocyte enhancer factor 2C | Mef2 |
| 17392 | Mmp3 | matrix metallopeptidase 3 | SL-1; EMS-2; SLN-1; STR-1; Stmy1 |
| 17700 | Mstn | myostatin | Cmpt; Gdf8 |
| 17885 | Myh8 | myosin, heavy polypeptide 8, skeletal muscle | MHCp; Myhsp;  Myhs-p; MyHC-pn |
| 14815 | Nr3c1 | nuclear receptor subfamily 3, group C, member 1 | GCR; Grl1; Grl-1 |
| 18221 | Nudc | nudC nuclear distribution protein | SIG-92; Silg92 |
| 18479 | Pak1 | p21 protein (Cdc42/Rac)-activated kinase 1 | Paka; PAK-1 |
| 18534 | Pck1 | phosphoenolpyruvate carboxykinase 1, cytosolic | PEPCK; Pck-1 |
| 66853 | Pnpla2 | patatin-like phospholipase domain containing 2 | Atgl; TTS-2.2 |
| 216151 | Polrmt | polymerase (RNA) mitochondrial | Q8BKF1 |
| 19130 | Prox1 | prospero homeobox 1 | P48437 |
| 54720 | Rcan1 | regulator of calcineurin 1 | CSP1; DSC1; RCN1; Dscr1; MCIP1; CALP1L; Adapt78 |
| 20208 | Saa1 | serum amyloid A 1 | Saa2; Saa-1 |
| 20716 | Serpina3n | serine peptidase inhibitor, clade A, member 3N | Spi2-2; Spi2.2; Spi2/eb.4 |
| 67712 | Slc25a37 | solute carrier family 25, member 37 | Mfrn; Mscp; Mfrn1; frascati; mitoferrin |
| 12702 | Socs3 | suppressor of cytokine signaling 3 | Cis3; Ef10; Ssi3; Cish3; EF-10; SSI-3 |
| 20848 | Stat3 | signal transducer and activator of transcription 3 | Aprf |
| 21422 | Tfcp2 | transcription factor CP2 | CP2; LSF; CP-2; LBP1; UBP-1; LBP-1c; LBP-1d; Tcfcp2; |
| 21846 | Tie1 | tyrosine kinase with immunoglobulin-like and EGF-like domains 1 | TIE; tie-1 |
| 433766 | Trim63 | tripartite motif-containing 63 | RF1; MuRF1; Rnf28 |
| 22084 | Tsc2 | tuberous sclerosis 2 | Tcs2; Nafld |
| 22142 | Tuba1a | tubulin, alpha 1A | Tuba1; Tuba-1 |
| 22145 | Tuba4a | tubulin, alpha 4A | M[a]4; Tuba4 |
| 22229 | Ucp3 | uncoupling protein 3 | UCP-3 |
| 22230 | Ufd1 | ubiquitin fusion degradation 1 like | UB |
