# Supplementary material for: The Pathway to Cancer Cachexia: MicroRNA-Regulated Networks in Muscle Wasting Based on Integrative Meta-Analysis
Source: Int J Mol Sci. 2019 Apr 22;20(8):1962. doi: 10.3390/ijms20081962 (PMC6515458; doi:10.3390/ijms20081962)
Supplement: Supplementary file 1 [file ijms-20-01962-s001.zip › S2 Table.docx]

**Supporting Material**

**S2 Table. Predicted and validated microRNAs.**

Number of predicted and validated microRNA-mRNA interactions

|  | **microRNAs (n)** | | | |
| --- | --- | --- | --- | --- |
| **mRNA Target** | **Predicted** | **Validated** | **Soares et al. 2014;**  **Narasimhan et al. 2017** | **Total** |
| Actc1 | 36 | 1 | 0 | 37 |
| Adcy7 | 87 | 0 | 0 | 87 |
| Adipoq | 37 | 0 | 0 | 37 |
| Angptl7 | 12 | 5 | 0 | 17 |
| Apcdd1 | 61 | 7 | 0 | 68 |
| Bnip3 | 29 | 1 | 0 | 30 |
| C1s1 | 12 | 0 | 0 | 12 |
| Camk2b | 108 | 2 | 0 | 110 |
| Cav1 | 66 | 6 | 1 | 73 |
| cEBP | 37 | 0 | 0 | 37 |
| Comp | 17 | 5 | 0 | 22 |
| Cxcl12 | 197 | 8 | 2 | 207 |
| Eif3i | 1 | 0 | 0 | 1 |
| Fap | 37 | 0 | 0 | 37 |
| Fbxo32 | 57 | 2 | 0 | 59 |
| Fgg | 28 | 0 | 0 | 28 |
| Foxo1 | 133 | 4 | 1 | 138 |
| Fst | 48 | 1 | 0 | 49 |
| Gabarapl1 | 45 | 0 | 0 | 45 |
| Hgs | 66 | 0 | 0 | 66 |
| Hint3 | 28 | 0 | 0 | 28 |
| HP | 35 | 0 | 0 | 35 |
| Hsd11b1 | 69 | 1 | 0 | 70 |
| Hsp90ab1 | 62 | 0 | 0 | 62 |
| Junb | 76 | 0 | 1 | 77 |
| Kcnip4 | 52 | 0 | 0 | 52 |
| Lama2 | 20 | 0 | 0 | 20 |
| Mef2c | 33 | 14 | 2 | 49 |
| Mmp3 | 40 | 0 | 0 | 40 |
| Mstn | 65 | 0 | 0 | 65 |
| Myh8 | 26 | 0 | 0 | 26 |
| Nr3c1 | 50 | 9 | 0 | 59 |
| Nudc | 38 | 0 | 0 | 38 |
| Pak | 60 | 2 | 0 | 62 |
| Pck1 | 86 | 5 | 0 | 91 |
| Pnpla2 | 66 | 1 | 0 | 67 |
| Polrmt | 9 | 0 | 0 | 9 |
| Prox1 | 62 | 2 | 0 | 64 |
| Rcan1 | 85 | 1 | 0 | 86 |
| Saa1 | 44 | 0 | 0 | 44 |
| Serpina3n | 139 | 0 | 0 | 139 |
| Slc25a37 | 133 | 2 | 0 | 135 |
| Socs3 | 75 | 1 | 0 | 76 |
| Stat3 | 124 | 9 | 0 | 133 |
| Tfcp2 | 78 | 9 | 0 | 87 |
| Tie1 | 50 | 0 | 0 | 50 |
| Trim63 | 121 | 0 | 0 | 121 |
| Tsc2 | 99 | 0 | 0 | 99 |
| Tuba1a | 32 | 0 | 0 | 32 |
| Tuba4a | 97 | 0 | 0 | 97 |
| Ucp3 | 82 | 0 | 0 | 82 |
| Ufd1 | 0 | 0 | 0 | 0 |
| **TOTAL** | **3150** | **98** | **7** | **3255** |
