# Supplementary material for: The Pathway to Cancer Cachexia: MicroRNA-Regulated Networks in Muscle Wasting Based on Integrative Meta-Analysis
Source: Int J Mol Sci. 2019 Apr 22;20(8):1962. doi: 10.3390/ijms20081962 (PMC6515458; doi:10.3390/ijms20081962)
Supplement: Supplementary file 1 [file ijms-20-01962-s001.zip › S3 Table.docx]

**Supporting Material**

**S3 Table. MicroRNA-mRNA interactions.**

List of predicted and validated microRNA-mRNA interactions

|  | **Target** | **Validated microRNA** |
| --- | --- | --- |
| ***Up-regulated*** | Stat3 | miR-106a-5p, miR-93-5p, miR-20a-5p, miR-17-5p, let-7b-5p, miR-125b-5p, miR-106b-5p, miR-223-3p, miR-124-3p |
|  | Tfcp2 | miR-3089-5p, miR-3082-5p, miR-804, miR-759, miR-1195, miR-665-3p, miR-673-3p, miR-5135, miR-139-5p |
|  | Cxcl12 | miR-124-3p, miR-140-5p, miR-17-5p, miR-340-5p, miR-27b, miR-15a-5p, miR-34b-5p, miR-149-5p, miR-9-5p |
|  | Angptl7 | miR-1195, miR-804, miR-3089-5p, miR-5135, miR-376c-3p, |
|  | Comp | miR-329-3p, miR-362-3p, miR-100-5p, miR-99a-5p, miR-99b-5p |
|  | Foxo1 | miR-139-5p, miR-145a-5p, miR-694, miR-27a, miR-1264-3p |
|  | Pck1 | miR-342-3p, miR-377-3p, miR-362-3p, miR-329-3p, miR-466i-3p |
|  | Camk2b | miR-122-5p, miR-136-5p |
|  | Fbxo32 | miR-3098-5p, miR-384-3p |
|  | Pak1 | miR-425-5p, miR-34b-5p |
|  | Pnpla2 | miR-124-3p |
|  | Socs3 | miR-483-5p |
|  | Mstn | miR-27b |
|  | Junb | miR-199a |
| ***Under- regulated*** | Mef2c | miR-223-3p, miR-24-3p, miR-26a-5p, miR-27a, miR-27b, miR-327, miR-495-3p, miR-1192, miR-5101, miR-106b-5p, miR-20a-5p, miR-20b-5p, miR-93-5p, miR-17-5p, miR-106a-5p |
|  | Nr3c1 | miR-425-5p, miR-362-5p, miR-30e-5p, miR-129-5p, miR-1897-5p, miR-3057-5p, miR-5130, miR-28b, miR-28c |
|  | Cav1 | miR-340-5, miR-301-3p, miR-17-5p, miR-19b-3p, miR-199a, miR-297-5p, miR-203-3p |
|  | Apcdd1 | miR-301b-3p, miR-26a-5p, miR-3968, miR-3971, miR-1955-3p, miR-690 |
|  | Slc25a37 | miR-362-5p, miR-3082-3p |
|  | Prox1 | miR-181a-5, miR-124-3p |
|  | Rcan1 | miR-122-5p |
|  | Bnip3 | miR-221-3p |
|  | Hsd11b1 | miR-26a-5p |
|  | Fst | miR-124-3p |
|  | Actc1 | miR-124-3p |
